# 18q Deletion Syndrome Presenting with Late-Onset Combined Immunodeficiency

**DOI:** 10.1007/s10875-024-01751-4

**Published:** 2024-06-19

**Authors:** Sho Hashiguchi, Dan Tomomasa, Takuro Nishikawa, Shuji Ishikawa, Harumi Akaike, Hidehiko Kobae, Tsuyoshi Shirai, Toshikage Nagao, Kosuke Noma, Satoshi Okada, Kazuhiro Kamuro, Yasuhiro Okamoto, Hirokazu Kanegane

**Affiliations:** 1 Department of Pediatrics, Yamabiko Medical Welfare Center, Kagoshima, Japan; 2 Department of Pediatrics and Developmental Biology, Graduate School of Medical and Dental Sciences, Tokyo Medical and Dental University (TMDU), Tokyo, Japan; 3 Department of Pediatrics, Graduate School of Medical and Dental Sciences, Kagoshima University, 8-35-1 Sakuragaoka, Kagoshima City, 890-8520 Japan; 4 Department of Respiratory Medicine, Graduate School of Medical and Dental Sciences, Tokyo Medical and Dental University (TMDU), Tokyo, Japan; 5 Department of Hematology, Graduate School of Medical and Dental Sciences, Tokyo Medical and Dental University (TMDU), Tokyo, Japan; 6 Department of Pediatrics, Graduate School of Biomedical and Health Sciences, Hiroshima University, Hiroshima, Japan; 7 Department of Child Health and Development, Graduate School of Medical and Dental Sciences, Tokyo Medical and Dental University (TMDU), 1-5-45 Yushima, Bunkyo-ku, Tokyo, 113-8519 Japan

**Keywords:** Array-based comparative genomic hybridization, Common variable immunodeficiency, Late-onset combined immunodeficiency, *Pneumocystis* pneumonia, 18q deletion syndrome

## Abstract

Patients with chromosome 18q deletion syndrome generally experience hypogammaglobulinemia. Herein, we describe two patients with chromosome 18q deletion syndrome who presented with late-onset combined immune deficiency (LOCID), which has not been previously reported. Patient 1 was a 29-year-old male with 18q deletion syndrome, who was being managed for severe motor and intellectual disabilities at the Yamabiko Medical Welfare Center for 26 years. Although the patient had few infections, he developed *Pneumocystis* pneumonia at the age of 28. Patient 2, a 48-year-old female with intellectual disability and congenital malformations, was referred to Tokyo Medical and Dental University Hospital with abnormal bilateral lung shadows detected on her chest radiography. Computed tomography showed multiple lymphadenopathies and pneumonia. A lymph node biopsy of the inguinal region revealed granulomatous lymphadenitis, and a chromosomal examination revealed 18q deletion. Array-based genomic hybridization analysis revealed deletion at 18q21.32-q22.3 for patient 1 and at 18q21.33-qter for patient 2. Immune status work-up of the two patients revealed panhypogammaglobulinemia, decreased number of memory B cells and naïve CD4^+^ and/or CD8^+^ cells, reduced response on the carboxyfluorescein diacetate succinimidyl ester T-cell division test, and low levels of T-cell receptor recombination excision circles and Ig κ-deleting recombination excision circles. Consequently, both patients were diagnosed with LOCID. Although patients with 18q deletion syndrome generally experience humoral immunodeficiency, the disease can be further complicated by cell-mediated immunodeficiency, causing combined immunodeficiency. Therefore, patients with 18q deletion syndrome should be regularly tested for cellular/humoral immunocompetence.

**Supplementary Information:**

The online version contains supplementary material available at 10.1007/s10875-024-01751-4.

## Introduction

Chromosome 18q deletion syndrome (OMIM 601,808) results from the loss of a fragment from the long (q) arm of chromosome 18. It was first described in 1964 by de Grouchy et al. [1], and its overall incidence rate is approximately one in 40,000–55,000 [1, 2]. The syndrome presents with multiple congenital anomalies such as psychomotor developmental delay, hypotonia, short stature, hearing impairment, cleft palate, and congenital heart disease. Its prognosis is good in cases without complications such as severe infections and congenital heart disease. Patients with distal 18q deletions often present with autoimmune disorders and allergies. This phenotype also increases an individual’s susceptibility to various infections [2]. Approximately 50–90% of patients present with a decrease in at least one type of immunoglobulin (humoral immunodeficiency) and 32% present with a common variable immunodeficiency (CVID)-like phenotype [2]. In this study, we encountered two patients with chromosome 18q deletion syndrome who presented with late-onset combined immune deficiency (LOCID), which has not yet been reported in patients with the syndrome.

## Case Description

**Fig. 1:**
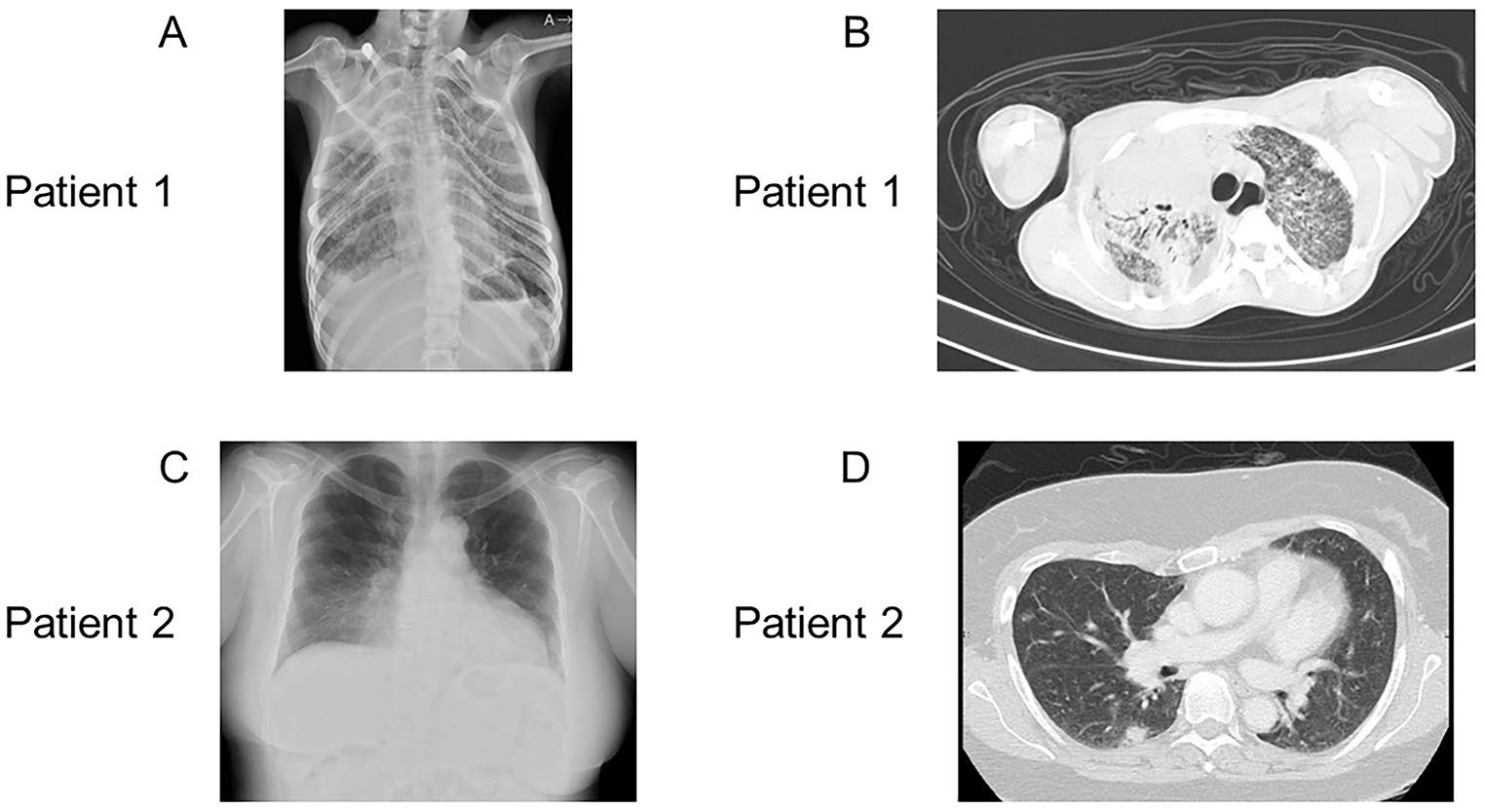
Radiological findings. **A**: (Patient 1) Chest X-ray revealing atelectasis in the right upper lobe. The right costophrenic angle is dull, with a granular shadow in the entire lung field. **B**: (Patient 1) Chest CT, showing slight compression of the right upper bronchus, presence of an air bronchogram in the right upper lobe, and consolidations in the left lung, which are believed to be inflammatory changes, suggesting that the patient has pneumonia. Granular shadows are observed in the lungs. **C**: (Patient 2) Chest X-ray showing abnormal bilateral lung shadows. **D**: (Patient 2) Chest CT showing mottled frosted shadows and irregular nodular shadows in both lungs

**Fig. 2:**
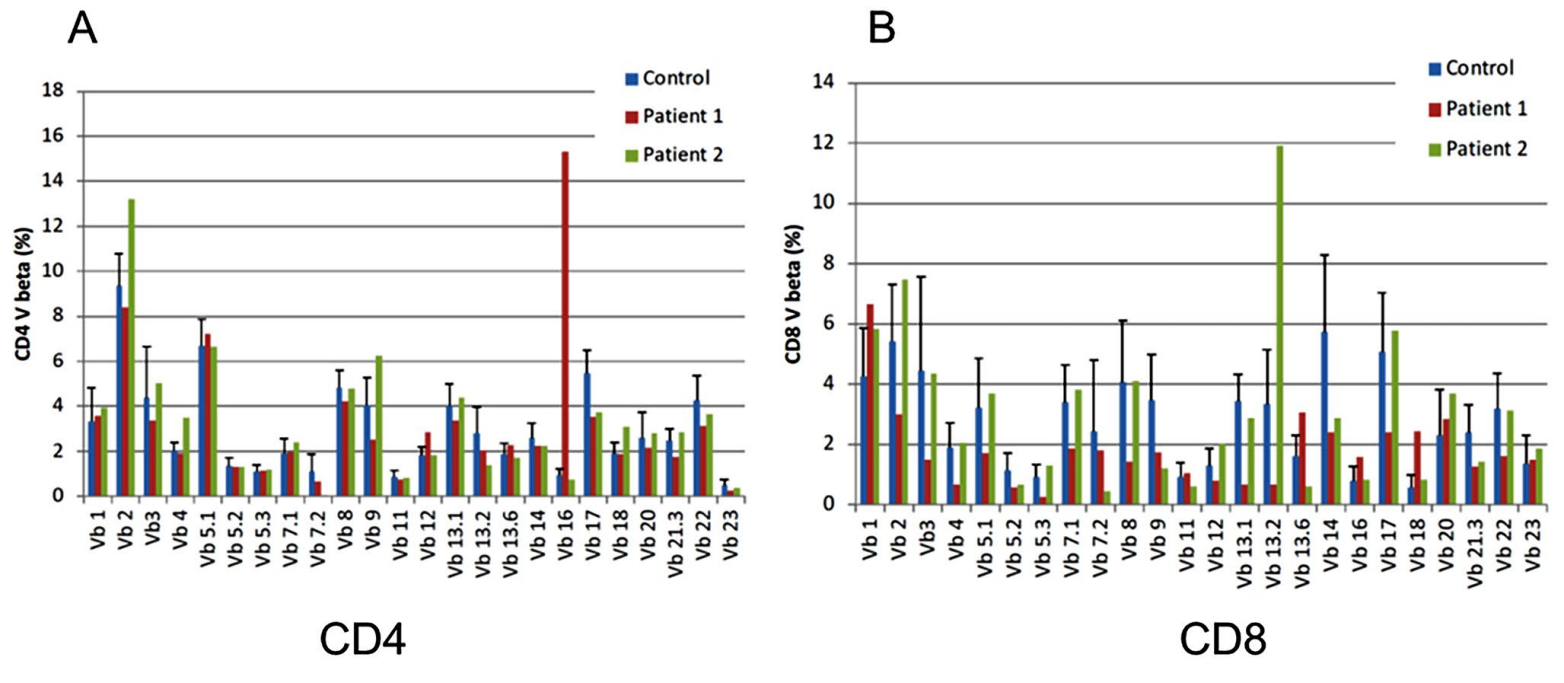
T-cell receptor Vb repertoire analysis. **A**: TCRVβ analysis of CD4^+^ T cells. The blue, red, and green bars indicate Vβ-positive cells in the T cells from the controls, Patient 1, and Patient 2, respectively. **B**: TCRVβ analysis of CD8^+^ T cells. The blue, red, and green bars indicate Vβ-positive cells in the T cells from the controls, Patient 1, and Patient 2, respectively

**Fig. 3:**
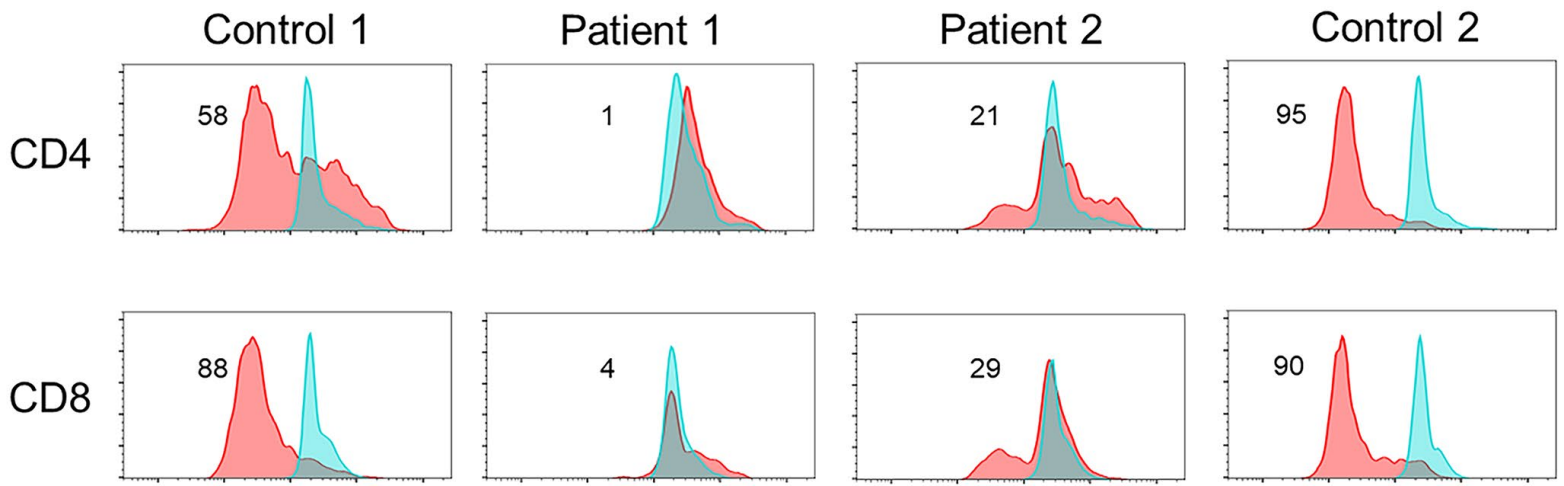
Results of T-cell proliferation assay. In the CFSE (carboxyfluorescein diacetate succinimidyl ester) T-cell proliferation test, CD4^+^ and CD8^+^ T lymphocytes do not undergo cell divisions in response to stimulus with phytohemagglutinin and are not activated compared to the control group. The blue and red shades indicate unstimulated and stimulated statuses, respectively. The percentages of cells after the application of stimuli are noted in the box

**Fig. 4:**
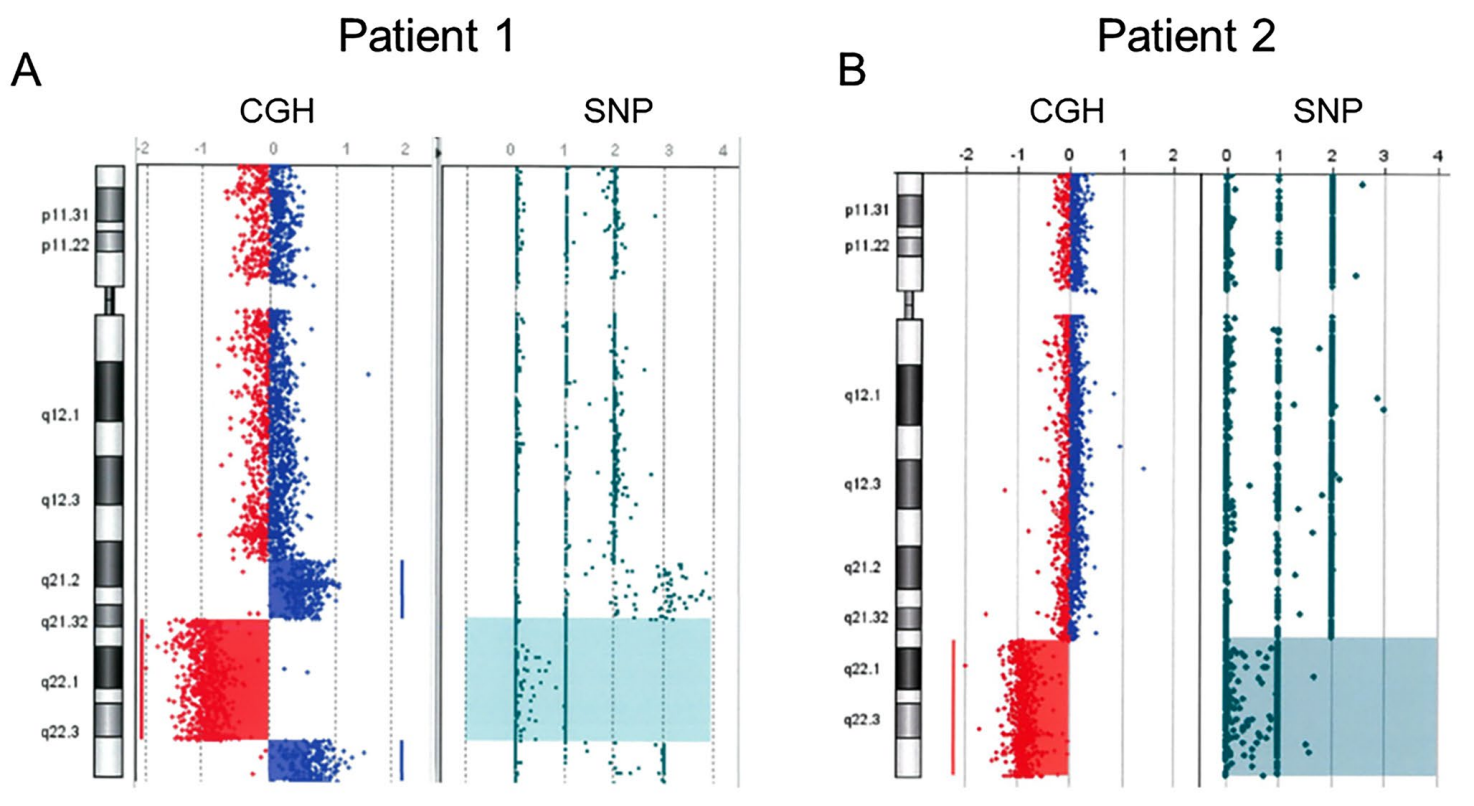
Results of comparative genomic hybridization and single-nucleotide polymorphism microarray analysis. The results of the CGH+SNP microarray for chromosome 18 show deletion from 18q21.32 to 18q22.3 (**A**: patient 1), and from 18q21.33 to 18qter (**B**: patient 2)

Patient 1 was a 29-year-old male with no family history of immunodeficiency or congenital anomalies. The patient was identified as having a complex chromosomal abnormality (46, XY, 10p+, 18q−, 20p−, 21q+), and was accordingly diagnosed with chromosome 18q deletion syndrome. Subsequently, the patient developed psychomotor developmental delays, epilepsy, and scoliosis. He was admitted to the Yamabiko Medical Welfare Center, Kagoshima, Japan with severe psychomotor developmental delays at 3 years of age. After admission, the patient did not experience infections. The measles–rubella combined vaccine, Bacillus Calmette–Guérin vaccine, and other vaccines were administered, and the patient presented no severe adverse reactions. At 27 years of age, he presented with a persistent fever and cough and was diagnosed with pneumonia following chest radiography and computed tomography (CT). Although various intravenous antibiotics and micafungin were administered, they were ineffective. Three months later, his β-D-glucan was found to be high (122 pg/mL; normal, ≤ 20 pg/mL), and he was diagnosed with *Pneumocystis* pneumonia (PCP) based on a *Pneumocystis jirovecii* PCR examination of his sputum. A combination of sulfamethoxazole and trimethoprim (ST) was administered and his symptoms improved rapidly. Six months after the resolution of the first episode of pneumonia, he developed a fever and cough, his percutaneous oxygen saturation decreased, and his chest X-ray and chest CT scans showed atelectasis in the right upper lobe and a granular shadow in all the lung fields (Fig. 1A and B). The patient was diagnosed with acute pneumonia. Although his β-D-glucan, aspergillus antigen, and candida antigen levels were normal (10 pg/mL, < 0.1 UA/mL, and < 0.1 UA/mL, respectively), the patient was suspected to have fungal pneumonia. Other opportunistic infections, such as herpes zoster, persistent intestinal viral infections, and herpes viral infections, were clinically ruled out. Micafungin was administered intravenously, and the drug combination ST was administered orally, which relieved his fever and symptoms. However, owing to the recurrent opportunistic infections, we decided to evaluate the patient’s immune status. His white blood cell count was 14,700/µL, with 70% granulocytes and 22% lymphocytes (3,263/µL). His serum IgG, IgA, and IgM levels were low (188, 105, and 26 mg/dL, respectively; normal ranges: 870–1,700, 110–410, and 33–190 mg/dL, respectively). His serum IgE level was < 5 IU/mL (normal value: $\le$232 IU/mL). Human immunodeficiency virus (HIV) antibody test results were negative. In the analysis of lymphocyte subpopulations (Table 1), CD3^+^ T cells comprised 71.3% of the total lymphocytes, NK cells comprised 18.1%, and CD19^+^ B cells decreased to 0.72%. Although CD4^+^ T cells were within the normal range, naïve CD4^+^ T cells were depleted, accounting for only 3.58% of the CD3^+^CD4^+^ cell population. The naïve CD8^+^T cells were within normal ranges. T-cell receptor (TCR)Vβ repertoire analysis revealed prominent skewing to Vβ16 for the CD4^+^ T cells (Fig. 2A). In the lymphocyte stimulation test, proliferation in response to both phytohemagglutinin (PHA) stimulation and concanavalin A was low (8,226 cpm; normal value: 20,500–56,800 cpm and 8,053 cpm; normal value: 20,300–65,700 cpm, respectively). The level of T-cell receptor recombination excision circles (TREC) was extremely low (25.27 copies/10^5^ cells, normal > 565 copies/10^5^ cells). Additionally, the Ig κ-deleting recombination excision circle (KREC) level was extremely low (93.36 copies/10^5^ cells; normal, ≥ 456 copies/10^5^ cells). The carboxyfluorescein diacetate succinimidyl ester (CFSE) cell proliferation test showed that the CD4^+^ and CD8^+^ T cells failed to divide in response to PHA (Fig. 3). T-cell proliferation studies were performed when the patient was not experiencing acute illness. Based on these findings, the patient was diagnosed with LOCID, a combination of humoral and cell-mediated immunodeficiencies. 18q deletion was confirmed through array-based comparative genomic hybridization (CGH) analysis, and presented with a loss of 18q21.32–q22.3 in the patient (Fig. 4A and Supplementary Table 1). The patient exhibited no morbid variants in his normal alleles without deletion of 18q in the whole-exome analyses. Subsequently, immunoglobulins were substituted periodically, and ST was administered prophylactically. However, the patient died of fungal pneumonia 2 years after the first episode.

**Table 1:**
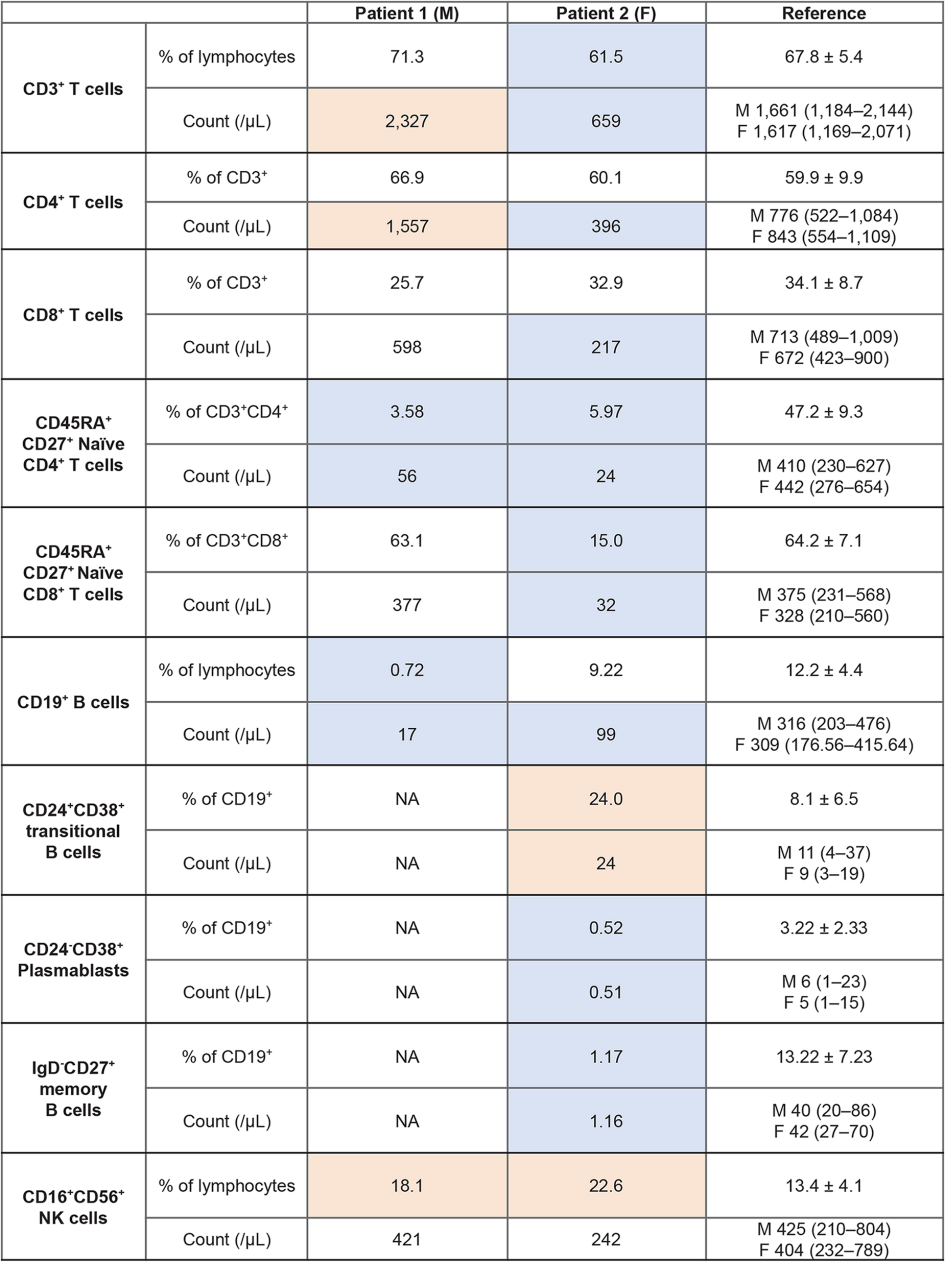
Lymphocyte subpopulations of patients 1 and 2

F, female; M, male; NK, natural killer; NA, not available data. Higher-than-reference values are highlighted in red and lower-than-reference values are highlighted in blue

Patient 2 was a 48-year-old female who was referred to the Tokyo Medical and Dental University Hospital, Tokyo, Japan with abnormal bilateral lung shadows, found during chest radiography at a routine annual checkup (Fig. 1C). She experienced intellectual disability, bilateral foot deformities, and bilateral third finger morphological abnormalities. She had been attending Tokyo Medical and Dental University Hospital because of hearing loss, congenital stenosis of the external auditory canal, and cleft lip and palate. Regarding her family history, no immune deficiencies or congenital anomalies were found. Chest CT revealed mottled frosted shadows and irregular nodular shadows in both lungs (Fig. 1D), as well as enlarged left supraclavicular fossa, bilateral axillae, mediastinum, abdominal cavity, and inguinal lymph nodes. A left inguinal lymph node biopsy was performed to differentiate lymphoproliferative diseases such as lymphoma. Consequently, the patient was diagnosed with granulomatous lymphadenitis and was followed up without treatment. G-banding of the biopsied lymph nodes revealed a chromosome 18 long-arm defect. The deletion was located at q21 on chromosome 18. Therefore, the patient was diagnosed with chromosome 18q deletion syndrome. Her white blood cell count was 5,100/µL, with 69% granulocytes and 21% lymphocytes (1,071 /µL). Her serum IgG, IgA, and IgM levels were low (8, 9, and 131 mg/dL, respectively). Her serum IgE level was 0.3 IU/mL, and her human immunodeficiency virus antibody test results were negative. Analysis of the lymphocyte subpopulations (Table 1) indicated that CD3^+^ T, NK, and CD19^+^ B cells comprised 61.5, 22.6, and 9.22% of the total lymphocytes, respectively. CD4^+^ T-cell levels were decreased to 396/µL. Furthermore, the naïve CD4^+^ T cells were depleted, accounting for only 5.97% of the CD3^+^CD4^+^ cell population. CD8^+^ T-cell levels were decreased to 217/µL, and naïve CD8^+^ T cells were depleted to 15.0% of the CD3^+^CD8^+^ cell population. Among the CD19^+^B cells, the proportion of IgD^−^CD27^+^ (class-switched memory) B cells was extremely low (1.17% of CD19^+^ cells). TCRVβ repertoire analysis showed prominent skewing to Vβ13.2 for the CD8^+^ T cells (Fig. 2B). The levels of TREC were extremely low (0 copies/10^5^ cells). Additionally, the KREC level was extremely low (11.4 copies/10^5^ cells). The CFSE T-cell proliferation assay performed after the acute phase indicated that the CD4^+^ and CD8^+^ T cells did not divide in response to PHA stimulation (Fig. 3). Based on these findings, patient 2 was diagnosed with LOCID. 18q deletion was confirmed by array-based CGH analysis, and presented with a loss of 18q21.33–qter in the patient (Fig. 4B and Supplementary Table 2). Analysis of the targeted panel sequence for 400 genes related to inborn errors of immunity (IEI) using DNA from peripheral blood mononuclear cells indicated that the patient did not exhibit any morbid variants in her normal alleles (without deletion of 18q). Immunoglobulin replacement therapy and prophylactic ST were subsequently initiated.

## Discussion

The clinical manifestations of chromosome 18q deletion syndrome include psychomotor developmental delays (96%), hypotonia (89%), convulsions (22%), short stature (55%), hypoplasia of the midsection of the face (78%), strabismus (30%), ear anomaly (67%), hearing impairment (72%), aural atresia (4%), cleft lip and palate (67%), microcephaly (59%), limb skeletal deformities (85%), reproductive organ defects (hypoplasia, cryptorchid testes, micropenis) (30%), urinary tract malformation (19%), congenital heart diseases (48%), atopic dermatitis (22%), and hypoventilation [2, 3]. Patients with 18q deletion syndrome exhibit a deficiency in at least one of the immunoglobulins, IgA, M, G, E, and IgG_1 − 4_ in 88% of cases [2], with IgE deficiency being the most common (52%), followed by deficiencies in the IgG subclass (42%), IgM (40%), IgG (32%), and IgA (20%) [2]. Therefore, 18q deletion syndrome is recognized as a cause of hypogammaglobulinemia [2, 4, 5].

Table 2 presents the clinical features and laboratory findings of the two patients. Patient 1 presented with psychomotor developmental delays, hypotonia, short stature, hypoplasia of the midsection of the face, strabismus, ear anomalies, aural atresia, and cryptorchidism as diagnostic features. Patient 2 exhibited psychomotor developmental delays, ear anomalies, cleft lip and palate, and skeletal deformities of the limbs as diagnostic features. Approximately 94% of 18q deletion syndrome cases are *de novo* [3], and similarly, both cases in this study were *de novo*. The patients in the present study exhibited low levels of IgG, IgM, IgA, and IgE. Additionally, patients with this syndrome present with recurrent infections of the respiratory (37%), urinary (19%), and gastrointestinal (19%) tracts, as well as sepsis (11%), indicating signs of humoral immunodeficiency [2, 4, 5]. Patients with 18q deletions frequently experience autoimmune diseases, repetitive infections, and allergies due to immune dysregulation, and present with variable antibody and regulatory T cell deficiencies. Both patients were negative for HIV antibodies and not receiving any drugs for treatment, including antiepileptic drugs. Therefore, secondary immunodeficiency was unlikely.

**Table 2:** Clinical features and laboratory findings

	**Clinical features**		
	**Patient 1**	**Patient 2**	
Growth	Short stature		
Face	Aural atresia Ear malformation Microsomia in the middle of the face Strabismus	Cleft lip and palate Congenital stenosis of the external auditory canal	
Extremities		Congenital anomaly of foot and third finger	
Genitourinary system	Cryptorchidism		
Nervous system	Intellectual disability	Intellectual disability	
	Hypotonia	Hearing loss	
	**Laboratory data**		
	**Patient 1**	**Patient 2**	**Normal value**
WBC (µL)	14,700	5,100	4,400–9,100
Neutrophil (%)	70	69	30–40
Lymphocyte (%)	22	21	50–60
Monocyte (%)	8%	22.6%	1–4
PHA (cpm)	8,226	NA	41,000–79,900
ConA (cpm)	8,053	NA	34,400–62,300
IgG (mg/dl)	188	8	870–1,700
IgA (mg/dl)	105	9	110–410
IgM (mg/dl)	26	131	33190
IgE (IU/ml)	< 5	0.3	$\le$232
TREC (copies/10^5^ cells)	25.27	0	$>$565
KREC (copies/10^5^ cells)	93.36	11.4	≥ 456

NA; not assessed, PHA; phytohemagglutinin, ConA; concanavalin A, TREC; T-cell receptor recombination excision circles, KREC; Ig κ-deleting recombination excision circles

CVID is associated with a differentiation failure of memory B and plasma cells. CVID is the most common primary immunodeficiency in adulthood [6, 7]. LOCID is a type of CVID that is distinguished from CVID by the presence of T-cell abnormalities. CVID can be distinguished from combined immune deficiency according to TREC and KREC levels [8]; patients with LOCID have low TREC and KREC levels. This classification, based on TREC/KREC levels, correlates well with complications, neoplasms, autoimmune diseases, and opportunistic infections, which are more common among patients with LOCID [8, 9]. Extremely low TREC and KREC levels were observed in the cases presented in the present study.

Patient 1 presented with a loss of 18q21.32-q22.3 on the CGH array, whereas patient 2 presented with a loss of 18q21.33-qter. In most patients with 18q deletion syndrome, the deletions are terminal and are localized in the distal half of the long arm (18q21.1-qter) [10], as observed for the patients in this study. Some genes (*NEDD4L, MALT1, TNFRSF11A, BCL2, CD226, SOCS6*, and *NFATC1* in patient 1, and *BCL2, CD226, SOCS6*, and *NFATC1* in patient 2) (Supplementary Tables 1, 2) that may be involved in IEI, were present at the chromosomal deletion sites of the patients. *MALT1* has an intrinsic T-cell role in regulating homeostasis; patients with homozygous missense variants in *MALT1* have severely impaired T-cell proliferation in response to antigens and antibodies, resulting in a combined immunodeficiency [11]. However, whole-exome analyses showed that patient 1 did not exhibit any morbid variants in the normal alleles (without the deletion of 18q). *TNFRSF11A* is essential for the correct differentiation of medullary thymic epithelial cells. Moreover, haploinsufficiency of *TNFRSF11A* and *NEDD4L* leads to a decrease in regulatory T cells [2, 12, 13]. *BCL2* is involved in immunoglobulin synthesis (variants that could cause CVID) and lymphocyte development. *CD226* is a signaling molecule that induces cytotoxic activity in T and NK cells. *SOCS6* is involved in lymphocyte maturation, and *NFATC1* is involved in maintaining regulatory T cell functions [2, 14, 15]. However, none of these genes act in an autosomal-dominant manner. There may be some unidentified genes that correlate with LOCID at sites where 18q is deleted or at other sites with deletions. Although there are many cases involving deletion of the same region as that of the two patients presented in this study, there are no reports of patients with 18q deletion syndrome developing LOCID. It is possible that these patients simply have not yet developed LOCID or that they might not have been adequately assessed for it. Patient 1 had a 21q + abnormality observed through G-banding. *DSCR1* is a gene suggested to contribute to the immunodeficiency observed in a proportion of patients with trisomy 21 [16]. The results of the CGH + SNP microarray for chromosome 21 of patient 1 showed deletion of 21q22.3 (Supplementary Fig. 1) and no *DSCR1* gene in the deleted region.

In IEI, susceptible infections and pathogens are characterized according to the type of immunodeficiency, whereas the putative primary immune deficiencies differ according to the type of infection [17]. Patients with antibody deficiency are susceptible to recurrent bacterial respiratory infections, whereas patients with cell-mediated immunodeficiencies are susceptible to opportunistic infections such as PCP. The drug combination ST is required to prevent PCP in patients with cell-mediated immunodeficiency; therefore, this drug combination is also administered orally to patients after a confirmed diagnosis. Periodic substitution of immunoglobulins is used to treat patients with hypogammaglobulinemia. Although adaptations for allogeneic hematopoietic cell transplantation could be considered for patients with LOCID, Patient 1 is believed to have had difficulty adapting to transplantation because of his general condition. Patient 2 would be considered for transplantation during the follow-up.

Patient 2 had not been diagnosed with the 18q deletion syndrome earlier. 18q deletion syndrome is associated with characteristic malformations; therefore, it should have been suspected, and chromosome testing should have been performed. Patient 1 was diagnosed with 18q deletion syndrome earlier but was not tested for immunocompetence. The occurrence of these two cases suggests that patients with 18q deletion syndrome should be tested for cellular/humoral immunocompetence regularly, at least once a year. When encountering patients with PCP, immunocompetent workups should be conducted, and attention should be paid to the fact that LOCID, the most severe immunodeficiency, can affect patients with 18q deletion syndrome. Although patients with 18q deletion syndrome are known to develop CVID, there is no recognition that they might develop LOCID; therefore, physicians should be aware of this aspect. The immune evaluation of these patients had several limitations. HIV antigen/PCR test was not performed, dihydrorhodamine 123 test was not performed for patient 1, antibody responses to pneumococcal serotypes were not assessed, and T-cell proliferation assays were performed only once.

In conclusion, to the best of our knowledge, we report the first diagnoses of two patients with chromosome 18q deletion syndrome who presented with LOCID. Our study highlights that patients with 18q deletion syndrome who present with recurrent or severe infections should be regularly tested for cellular/humoral immunocompetence.

### Electronic Supplementary Material

Below is the link to the electronic supplementary material.

Supplementary Material 1

## Data Availability

No datasets were generated or analysed during the current study.
