# Supplementary material for: 18q Deletion Syndrome Presenting with Late-Onset Combined Immunodeficiency
Source: J Clin Immunol. 2024 Jun 19;44(7):154. doi: 10.1007/s10875-024-01751-4 (PMC11186878; doi:10.1007/s10875-024-01751-4)
Supplement: Supplementary file 1 — Supplementary Material 1 [file 10875_2024_1751_MOESM1_ESM.docx]

*Original article*

**18q deletion syndrome presenting with late-onset combined immunodeficiency**

**Journal of Clinical Immunology**

Sho HASHIGUCHI^1†^, Dan TOMOMASA^2†^, Takuro NISHIKAWA^3*^, Shuji ISHIKAWA^1^, Harumi AKAIKE^1^, Hidehiko KOBAE^1^, Tsuyoshi SHIRAI^4^, Toshikage NAGAO^5^, Kosuke NOMA^6^, Satoshi OKADA^6^, Kazuhiro KAMURO^1^, Yasuhiro OKAMOTO^3^, Hirokazu KANEGANE^7^

^1^Department of Pediatrics, Yamabiko Medical Welfare Center, Kagoshima, Japan

^2^Department of Pediatrics and Developmental Biology, Graduate School of Medicine and Dental Sciences, Tokyo Medical and Dental University (TMDU), Tokyo, Japan

^3^Department of Pediatrics, Graduate School of Medical and Dental Sciences, Kagoshima University, Kagoshima, Japan

^4^Department of Respiratory Medicine, Graduate School of Medicine and Dental Sciences, Tokyo Medical and Dental University (TMDU), Tokyo, Japan

^5^Department of Hematology, Graduate School of Medicine and Dental Sciences, Tokyo Medical and Dental University (TMDU), Tokyo, Japan

^6^Department of Pediatrics, Graduate School of Biomedical & Health Sciences, Hiroshima University, Hiroshima, Japan

^7^Department of Child Health and Development, Graduate School of Medicine and Dental Sciences, Tokyo Medical and Dental University (TMDU), Tokyo, Japan

†These authors have contributed equally to this work and share the first authorship.

**Corresponding author:** Takuro Nishikawa, MD, PhD

Department of Pediatrics, Graduate School of Medical and Dental Sciences, Kagoshima University, 8-35-1 Sakuragaoka, Kagoshima City, 890-8520, Japan

Tel: +81-99-275-5354; Fax: +81-99-265-7196

E-mail: adu44150@ams.odn.ne.jp

**Supplementary Table 1. Microarray (CGH array) analysis showing the cytogenic location and genomic coordinates of gains, losses, and loss of heterozygosity (LOH) in chromosome 18 of Patient 1**

|  | Minimum start-stop (bp)  Maximum start-stop (bp) | Minimum cytoband  Maximum cytoband | Annotations |
| --- | --- | --- | --- |
| Gain | 50181426-57592641  50169630-57640896 | q21.2-q21.32  q21.2-q21.32 | DCC, MIR4528, LINC01919, LINC01917, MBD2, SNORA37, POLI, STARD6, C18orf54, DYNAP, RAB2​​7B, CCDC68, LINC01929, TCF4, TCF4-AS1, MIR4529, LINC01415, LINC01416, LOC642484, LINC01905, LINC01539, TXNL1, WDR7, LINC-ROR, BOD1L2, LINC02565, ST8SIA3, ONECUT2, FECH, NARS1, LOC100505549, ATP8B1, **NEDD4L**, MIR122, MIR3591, ALPK2, SNORA108, LOC101927322, **MALT1**, LINCO1926, ZNF532, OACYLP, SEC11C, GRP, RAX, CPLX4, LMAN1, CCBE1, PMAIP1 |
| Loss | 57640897-72836488  57592642-72882930 | q21.32-q22.3  q21.32-q22.3 | MC4R, CDH20, LINC01544, RNF152, PIGN, RELCH, **TNFRSF11A**, ZCCHC2, PHLPP1, **BCL2**, KDSR, VPS4B, SERPINB5, SERPINB12, SERPINB13, SERPINB4, SERPINB3, SERPINB11, SERPINB7, SERPINB2, SERPINB10, HMSD, SERPINB8, LINC00305, LINC01924, LINC01538, CDH7, CDH19, MIR5011, DSEL, LOC643542, LINC01903, TMX3, CCDC102B, DOK6, LOC105372179, **CD226**, RTTN, **SOCS6**, LINC01909, LIVAR, LINC01910, GTSCR1, LINC01541, LINC01899, CBLN2, NETO1, MIR548AV, LOC100505797, LINC02864, LINC02582, FBXO15, TIMM21, CYB5A, C18orf63, LINC01922, DIPK1C, CNDP2, CNDP1, LINC00909, ZNF407 |
| Gain | 72882931-78012829  72836489-78012829 | q22.3-q23  q22.3-q23 | ZADH2, TSHZ1, SMIM21, LINC01898, LOC339298, LINC01893, ZNF516, LOC101927989, ZNF516-DT, LINC00908, LINC00683, LINC01927, LINC01879, ZNF236-DT, ZNF236, MBP, GALR1, LINC01029, SALL3, ATP9B, **NFATC1**, LOC284240, LOC284241, CTDP1, KCNG2, SLC66A2, HSBP1L1, TXNL4A, RBFA, RBFADN, ADNP2, PARD6G- AS1, PARD6G |
| LOH | 57388443-72838019  57365625-72848598 | q21.32-q22.3  q21.32-q22.3 | PMAIP1, MC4R, CDH20, LINC01544, RNF152, PIGN, RELCH, TNFRSF11A, ZCCHC2, PHLPP1, **BCL2**, KDSR, VPS4B, SERPINB5, SERPINB12, SERPINB13, SERPINB4, SERPINB3, SERPINB11, SERPINB7, SERPINB2, SERPINB10, HMSD, SERPINB8, LINC00305, LINC01924, LINC01538, CDH7, CDH19, MIR5011, DSEL, LOC643542, LINCO1903, TMX3, CCDC102B, DOK6, LOC105372179, **CD226**, RTTN, **SOCS6**, LINC01909, LIVAR, LINC01910, GTSCR1, LINC01541, LINC01899, CBLN2, NETO1, MIR548AV, LOC100505797, LINC02864, LINC02582, FBXO15, TIMM21, CYB5A, C18orf63, LINC01922, DIPK1C, CNDP2, CNDP1, LINC00909, ZNF 407 |

The bold-underlined genes are known inborn errors of immunity located on 18q.

**Supplementary Table 2. Microarray (CGH array) analysis showing the cytogenic location and genomic coordinates of losses in chromosome 18 of**

**Patient 2**

|  | Minimum start-stop (bp)  Maximum start-stop (bp) | Minimum cytoband  Maximum cytoband | Annotations |
| --- | --- | --- | --- |
| Loss | 60453234-78012829  60430388-78012829 | q21.33-q23  q21.33-q23 | PHLPP1, **BCL2**, KDSR, VPS4B, SERPINB5, SERPINB12, SERPINB13, SERPINB4, SERPINB3, SERPINB11, SERPINB7, SERPINB2, SERPINB10, HMSD, SERPINB8, LINC00305, LINC01924, LINC01538, CDH7, CDH19, MIR5011, DSEL, LOC643542, LINC01903, TMX3, CCDC102B, DOK6, LOC105372179, **CD226**, RTTN, **SOCS6**, LINC01909, LIVAR, LINC01910, GTSCR1, LINC01541, LINC01899, CBLN2, NETO1, MIR548AV, LOC100505797, LINC02864, LINC02582, FBXO15, TIMM21, CYB5A, C18orf63, LINC01922, DIPK1C, CNDP2, CNDP1, LINC00909, ZNF407, ZADH2, TSHZ1, SHIM21, LINC01898, LOC339298, LINC01893, ZNF516, LOC101927989, ZNF516-DT, LINC00908, LINC00683, LINC01927, LINC01879, ZNF236-DT, ZNF236, MBP, GALR1, LINC01029, SALL3, ATP9B, **NFATC1**, LOC284240, LOC284241, CTDP1, KCNG2, SLC66A2, HSBP1L1, TXNL4A, RBFA, RBFADN, ADNP2, PARD6G-AS1, PARD6G |

The bold-underlined genes are known inborn errors of immunity located on 18q.


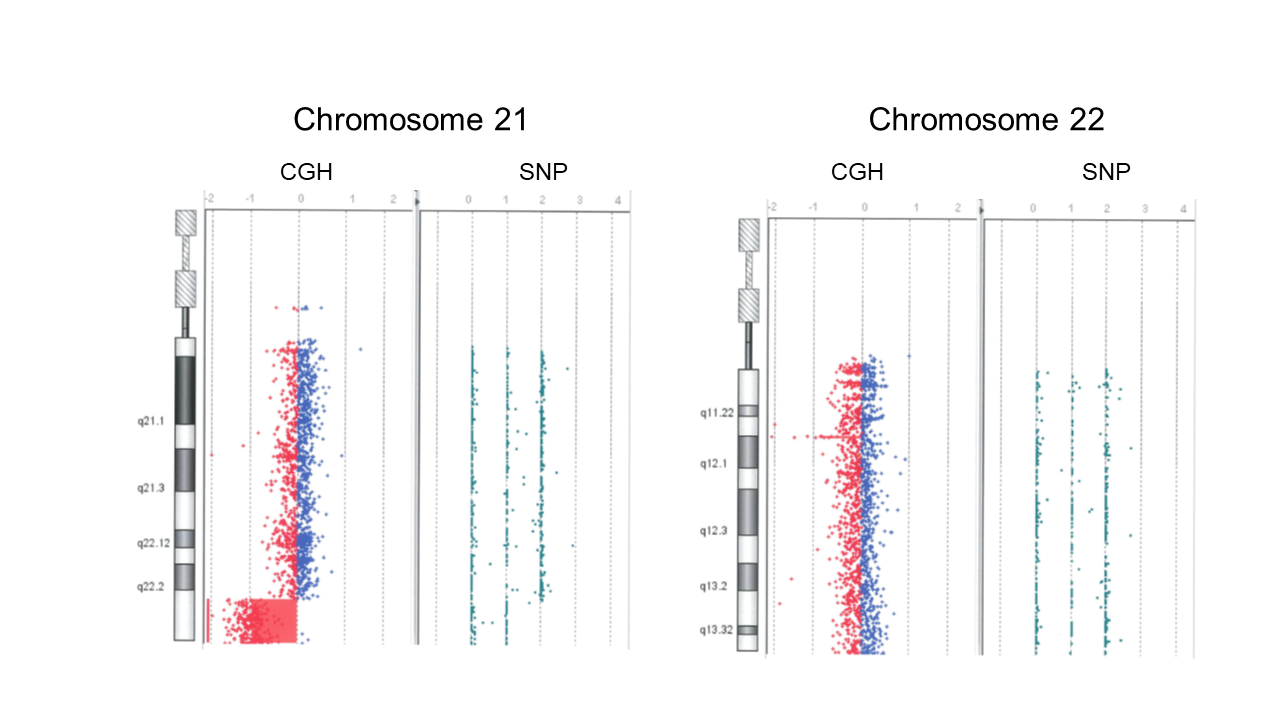


**Supplementary Fig. 1 Results of the comparative genomic hybridization (CGH) and single-nucleotide polymorphism (SNP) microarray analysis.**

The results of the CGH＋SNP microarray for chromosome 21 show deletion of 21q22.3.
